# Preparation and Application of Hydrophobic and Breathable Carbon Nanocoils/Thermoplastic Polyurethane Flexible Strain Sensors

**DOI:** 10.3390/nano15060457

**Published:** 2025-03-17

**Authors:** Yanming Sun, Yanchen Huang, Xiaoying Lu, Hao Song, Guoping Wang

**Affiliations:** 1 State Key Laboratory of Radio Frequency Heterogeneous Integration, Shenzhen University, Shenzhen 518060, China; 2 College of Electronics and Information Engineering, Shenzhen University, Shenzhen 518060, China; 3 Institute of Microscale Optoelectronics, Shenzhen University, Shenzhen 518060, China; 4 College of Physics and Electronic Information Engineering, Neijiang Normal University, Neijiang 641112, China

**Keywords:** hydrophobic, breathable, CNCs/TPU, flexible strain sensors

## Abstract

The emphasis on physical activity and health monitoring has increased the demand for developing multifunctional, flexible sensors through straightforward methods. A hydrophobic, breathable, and flexible strain sensor was prepared using a filtration method, employing thermoplastic polyurethane (TPU) as a substrate, carbon nanocoils (CNCs) as conductive fillers, and polydimethylsiloxane (PDMS) as a binder. The sensing layer, prepared using the unique three-dimensional helical structure of carbon nanocoils, achieved a hydrophobic angle of 143° and rapidly changed the color of the pH test paper in 5 s. The sensor had a strain range of 40% and a gauge factor of 34, and achieved a linear fit of R^2^ = 0.98 in the 5–35% strain range. The CNCs/TPU sensor exhibits high reliability and stability after 1000 tensile cycle tests. These favorable features ensure that the sensors are comfortable to wear and respond quickly and accurately to movements in all body parts, meeting the need for human motion detection.

## 1. Introduction

Increasing demand for daily activity monitoring and health detection has promoted the rapid development of smart wearable devices [1,2,3,4,5,6]. One of the most important wearable devices is the strain sensor, which converts externally generated signals of different physical deformations into recognizable electrical signals. Traditional sensors primarily use metals and semiconductors, which are hard and brittle, difficult to curl, breathable, uncomfortable, and have a limited monitoring range, making them unsuitable for some specific applications [7,8]. In contrast, flexible sensors have received more and more attention and research from scholars because of their excellent properties, such as their large tensile deformation, high sensitivity, softness and comfort, weightlessness, and fit to the body. Flexible sensors, characterized by their distinct properties, possess considerable potential for application in various domains, such as human motion monitoring, health monitoring, flexible electronic skin, and human–computer interaction [9,10,11,12,13,14].

Common flexible sensors mainly comprise a flexible substrate with tensile properties and a sensing layer with electrical conductivity. The flexible substrates are mostly polymers, such as cellulose [15], polyvinylidene fluoride (PVDF) [16], polydimethylsiloxane (PDMS) [17,18], thermoplastic polyurethane (TPU) [19,20,21], Ecoflex [22], natural rubber [23], and hydrogels [24]. The sensing layer primarily consists of nanometals [25,26,27], carbon nanomaterials [28,29,30], conductive polymers [31,32], and other materials.

Compared with the general chemical stability of nanometals, the devices have limited tensile resistance, poor mechanical properties, and poor stability of the conducting polymers. Carbon nanomaterials have great potential for sensing layers due to their excellent mechanical and electrical properties. CNCs have garnered particular attention due to their distinctive helical morphology. These unique properties have led to a variety of applications, such as electromagnetic wave absorption [33], electrochemical energy storage [34,35], and gas sensors [36], as well as applications in the nanorobotics field [37,38]. Li et al. [39] prepared an ultrasensitive strain sensor for detecting pulses by electrophoresis using CNCs as the sensing layer and PDMS as a substrate. Later, Yang et al. [40] further enhanced the detection range and sensitivity by growing CNT on the surface of CNCs. However, these sensors encapsulated with PDMS are not breathable, limiting their application in wearable strain sensors. When the sensor is attached to the skin for long durations, because the skin is breathable, using impermeable materials reduces the skin’s metabolic rate, leading to an itchy sensation [41]. In addition, secretions from sweat and sebaceous glands may trigger dermatitis, and sweat production also affects body signs. Therefore, the hydrophobic breathability of sensors is an issue that must be addressed to prolong wear and improve application suitability.

Wide linear sensing range and high sensitivity are the key performance indicators for the practical application of flexible strain sensors. Among them, the wide linear sensing range enables the sensor to accurately detect different degrees of stretch under large strains, and the high sensitivity reflects the sensor’s ability to respond to tensile strains. However, some challenges still persist in the current research on certain flexible strain sensors, such as the poor binding of conductive particles to the flexible polymer substrate, leading to poor cyclic stability during repeated loading [42,43]. Therefore, selecting materials and adjusting the preparation are the primary methods to solve the bonding problem between the sensing layer’s conductive particles and the substrate layer’s polymer, which is also the priority for preparing high-performance sensors.

Herein, a TPU fiber film with high stretchability and breathability was prepared using electrostatic spinning technology as a flexible substrate for wearable strain sensors. Thereafter, a vacuum filtration method was utilized to deposit the CNCs solution with PDMS onto the TPU fiber film, forming the stretchable strain sensor easily and quickly. The effect of the content of PDMS and CNCs on the performance of sensors was investigated by testing and characterizing the morphology, structure, electrical conductivity, air permeability, and sensing properties of the CNCs/TPU sensors. Additionally, the effect of the sensors on detecting human motion and pressure was discussed. The CNCs/TPU sensors were found to be soft, sensitive, stable, and to have a wide detection range, with up-and-coming application prospects in motion and pressure monitoring, electronic skin, and robotics.

## 2. Experimental Section

### 2.1. Materials

Thermoplastic polyurethane (TPU Elastollan 1185 A) was provided by BASF(Hürth, Germany). Polydimethylsiloxane (PDMS) was purchased from Dow Corning Corporation (Midland, MI, USA). Fe(NO_3_)_3_·9H_2_O, SnCl_4_·5H_2_O, N, N-dimethylformamide (DMF), tetrahydrofuran (THF), and n-pentane were obtained from Sinopharm Chemical Reagent Co. (Shanghai, China) Silver adhesive (Model No. 3701) was purchased from Shenzhen Sinwe New Material Co. (Shenzhen, China).

### 2.2. Synthesis of Carbon Nanocoils

The preparation of large quantities of CNC using CVD has been reported [44,45]. A total of 1 mmol of Fe^3+^ salt (Fe (NO_3_)_3_·9H_2_O) and 0.1 mmol of Sn^4+^ salt (SnCl_4_·5H_2_O) was weighed into a 36 mL beaker of N, N-dimethylformamide (DMF), sonicated for 30 min, then transferred into a microwave reactor to be heated for 3 h. After cooling to the right temperature, the catalyst was collected using a vacuum filter. The catalyst was dissolved in an ethanol solution at 1 mg/mL, then drop-coated onto an alumina ceramic sheet and dried in an oven. The catalyst-loaded ceramic sheet was placed in a chemical vapor deposition (CVD) apparatus. Argon (Ar) was introduced at a flow rate of 300 sccm for 30 min to purge air from the chamber. The temperature was then raised to 710 °C, after which acetylene (C_2_H_2_) was introduced at a flow rate of 33 sccm and maintained for 3 h to facilitate CNC growth. No other purge gas was used during the process. Finally, the CNCs were collected by scraping them from the ceramic sheet. The mass growth of CNCs and the TEM of a single CNC are shown in Figure S1 (seen in Supporting Information).

### 2.3. Preparation of TPU Electrospun Membrane

Firstly, the TPU particles were dissolved in a binary solvent system comprising DMF and THF (1:1 *v*/*v* ratio) under continuous magnetic stirring for 10 h, yielding a homogeneous 25 wt% polymer solution. The prepared solution was subsequently transferred to a syringe pump for electrospinning. A high voltage of 12.7 kV was applied between the metallic needle tip and the grounded collector to establish the electrostatic field. The solution was extruded at a controlled flow rate of 0.06 mL/min. Following a 90 min electrospinning process, the resultant non-woven TPU fibrous membrane was collected and was subsequently employed as a filtration substrate for CNC deposition.

### 2.4. Preparation of Flexible Strain Sensors Based on CNCs/TPU

**Figure 1:**
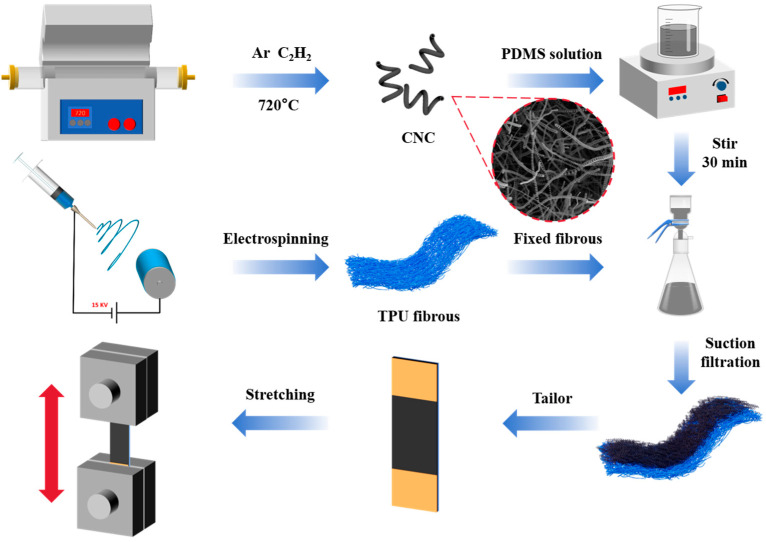
Preparation process diagram of flexible strain sensors based on CNCs/TPU.

A total of 2 mg of CNCs and different contents of PDMS (0.5 g, 1 g, and 2 g), together with the curing agent (10:1, *w*/*w*), were mixed in 30 mL of n-pentane solvent and were stirred for 30 min to obtain a uniform suspension. Then, the solution was dropped on the prepared TPU film, and the CNCs/PDMS were deposited uniformly on the TPU film using vacuum infiltration. The CNCs/TPU films were placed in an oven at 80 °C for 2 h to complete the curing of PDMS. The CNCs/TPU films were cut into 15 mm × 30 mm rectangles, coated with conductive silver adhesive on both sides of the edges, and bonded with conductive silver adhesive. Finally, the CNCs/TPU flexible strain sensors were obtained. Figure 1 shows this process.

### 2.5. Characterization

A field emission scanning electron microscope (FE-SEM, FEI Scios, Greifswald, Germany) was used to observe the morphological features. A JEOL 2200FS electron microscope with an operating voltage of 200 kV was used to observe the surface morphology of CNCs/TPU membrane composites. The surface superhydrophobicity was measured using an optical angle measuring device (OCA20, Dresden, Germany) to measure the water contact angle (WCA). Tensile testing and fatigue testing of the sensors were conducted using a microcomputer-controlled electronic universal testing machine (Meters Industrial Systems (China) Co. Ltd., Shanghai, China). A digital multimeter (DMM6500, Keithley, Cleveland, OH, USA) was used to record the real-time resistance changes of the material.

## 3. Results and Discussion

### 3.1. Effect of PDMS Content on the Sensing Layer

**Figure 2:**
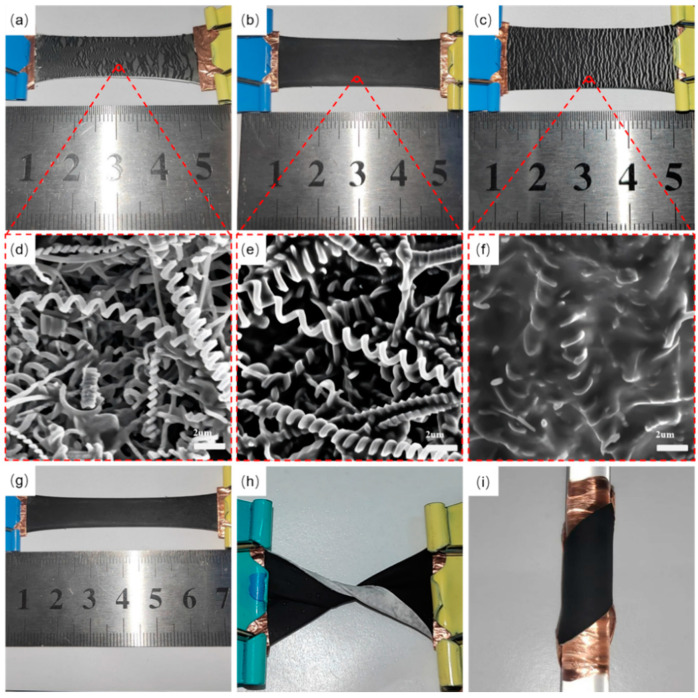
The sensors were prepared with 0.5 g (**a**), 1 g (**b**), and 2 g (**c**) PDMS content and stretched 100%. SEM images of sensors prepared with 0.5 g (**d**), 1 g (**e**), and 2 g (**f**) of PDMS content. Sensor with 1 g PDMS content (**g**) stretched 200%, (**h**) twisted 360°, and (**i**) wound around a glass rod.

The primary problem facing the preparation of sensors is bonding the sensing layer to the substrate, which relates to the sensing performance and durability of the sensor. Thus, the sensing layer was firmly connected to the substrate by adding CNCs into the diluted PDMS solution. To determine the optimal PDMS content in the solution, a mixture of 0.5 g, 1 g, and 2 g of PDMS with 2 mg of CNCs was prepared, and the morphology of the films was observed using SEM after being stretched to 100%. Figure 2 shows the film stretching and SEM for different PDMS contents.

In SEM imaging, the conductive part of the sample is usually brighter, and the non-conductive part is usually darker. As the CNCs are electrically conductive and PDMS is non-conductive, the adhesion of CNCs with different contents of PDMS can be assessed. Figure 2a shows that when the content of PDMS is 0.5 g and stretched to 100% of its original length, the sensing layer of CNCs is completely separated from the substrate and is detached. In Figure 2d, very little or even no attachment of PDMS exists on the surface of the CNCs, making the adhesion between the CNCs and the substrate very poor. Figure 2b shows stretching to the same length when the content of PDMS is 1 g. The CNC sensing layer does not change much with increased stretching and remains intact. Through the SEM of Figure 2e, it was observed that PDMS wrapped around the CNCs, making the CNCs tightly adhere to each other and to the substrate, which meets the preparation requirements of the sensor. Figure S2a,b shows a cross-section of the sensor prepared from 1 g PDMS. Figure 2c shows that when the content of PDMS is 2 g and the stretching is 100%, cracks are visible in the sensing layer due to the overly tight adhesion between PDMS and the substrate. Figure 2f also revealed that the CNCs were entirely covered by PDMS, making the sensing layer less recoverable after stretching. Figure S2c shows a cross-section of the sensor prepared with 2 g of PDMS.

As a result, a film with 1 g of PDMS content was selected for the stretching experiment. As shown in Figure 2g, the film with an original length of 2 cm was stretched to 200%, and the surface remained intact without peeling off. As shown in Figure 2h,i, when the film was twisted 360° and attached to a glass rod, the CNC sensing layer did not wrinkled and did not peel off, indicating that the CNC sensing layer stably attached to the TPU substrate.

**Figure 3:**
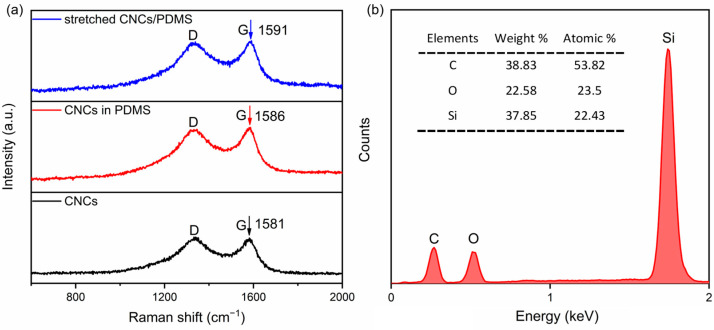
(**a**) Raman spectra of CNCs, CNCs wrapped in PDMS, and CNCs/PDMS in the stretched state. (**b**) EDS of the sensing layer on the sensor surface.

To verify that the CNCs/TPU sensors prepared using the extraction method and immobilized in PDMS maintain structural integrity during repeated stretching, the bonding strength of the CNCs to the PDMS was further investigated. Raman spectroscopy effectively evaluates the bond strength at the interface between the filler material/polymer in polymer composites. Figure 3a shows the Raman spectra of CNCs, CNCs/PDMS, and CNCs/PDMS in the stretched state. When the CNCs were encapsulated in PDMS, the G peak position moved from 1581 cm^−1^ to 1586 cm^−1^. The blueshift of the peak position originated from the change in the vibrational mode of the carbon atoms and the vibrational energy on the surface of the CNCs. When the sensor was stretched, the position of the G peak shifted from 1586 cm^−1^ to 1591 cm^−1^. The change in the distance between the carbon atoms in the CNC affected the vibrational state of the C-C bond, which shifted the peak’s position. The movement of the G peak indicates that the CNCs stretch together with stretching of the PDMS, which laterally indicates a strong bond between the two and lays the foundation for stabilizing the performance of the CNCs/TPU sensors. Figure 3b shows that the surface of the sensing layer is mainly composed of C, O, and Si elements, where Si:O is about 1:1, which also corresponds to the chemical formula of PDMS (C_2_H_6_OSi)_n_, indicating that the sensing layer is composed of a combination of PDMS and CNCs. In addition, Figure S3 shows the XPS analysis, which also demonstrates the simple elemental composition of the sensor and the absence of oxidation treatment, reflecting the stability of the material.

### 3.2. Characterization of the Sensor’s Performance

**Figure 4:**
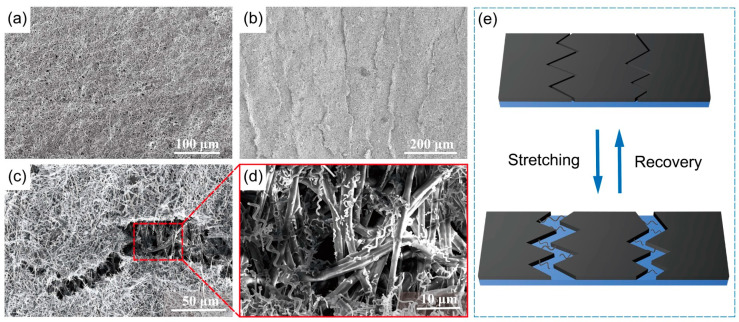
SEM image of sensors: (**a**) without stretching; (**b**) after stretching and recovery. (**c**,**d**) SEM images of cracks. (**e**) Schematic diagram of the crack formation process.

To better understand the sensing mechanism, the effect of stretching on the morphology of the sensing layer material was investigated. Figure 4a shows the SEM image of the sensor before stretching. It was observed that a uniform conductive layer of CNCs was attached to the surface of the TPU film, and many air holes were present on the surface of the conductive layer, which is conducive to the transmission of gases. As seen in Figure 4b, the sensing layer composed of CNCs remained tightly locked after the sensor was stretched and recovered, ensuring that the sensor’s resistance was stable in the subsequent resistance test after further stretching and recovery. Figure 4c,d depicts the magnified morphology of the cracks after stretching. The TPU on the crack was loaded with a few scattered, broken CNCs, and some larger, tough CNCs were on the edges. Figure S4 shows CNCs bridging both sides of the crack at small strains. The two parts ensure that the conductive CNCs can still be interconnected to form a conductive network when the sensor is stretched. Figure 4e shows the sensing principle. The sensor’s response is primarily driven by crack propagation and contact resistance changes. Under tensile loading, the widening of existing cracks and the formation of new cracks reduces the contact points between adjacent CNCs, increasing resistance. Upon unloading, the cracks partially close, reconnecting the CNCs and restoring conductivity. The helical structure of CNCs enhances mechanical interlocking with the TPU substrate, ensuring stable electron transport and consistent sensitivity even under repeated strain, thereby improving the sensor’s durability and performance in human motion detection.

The sensitivity of a sensor is an important parameter for evaluating its sensing performance and is represented by the slope of the strain–resistivity curve as the gauge factor (GF). To test the strain-sensing performance of a transducer, sensitivity should be calculated. The GF was calculated as follows:

(1) $$GF=\frac{∆R{/R}_{0}}{\varepsilon }$$

where R_0_ is the initial resistance of the sensor without strain, ΔR is the difference between the real-time resistance of the sensor and the initial resistance when strain is applied, and ε is the sensor strain, defined as ε = ΔL/L_0_, where ΔL is the displacement of the sensor under strain and L_0_ is the initial length of the sensor.

**Figure 5:**
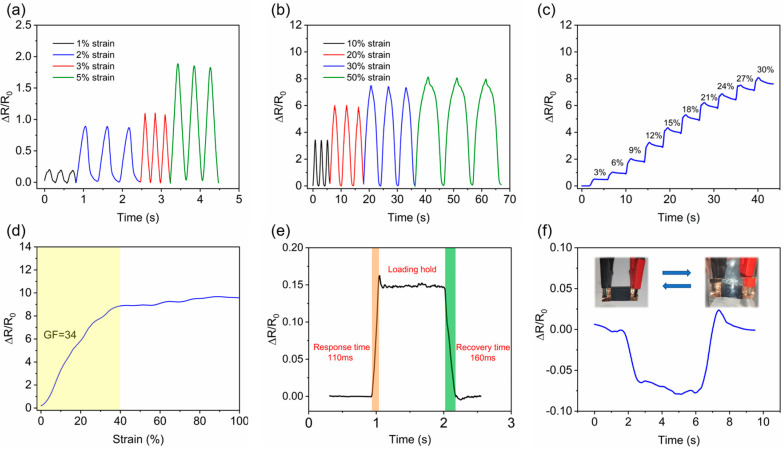
(**a**) Sensor tensile test at 0.1–0.5%, (**b**) sensor tensile test at 10–50%, (**c**) step test at 3% per stretch, (**d**) sensor sensitivity test(yellow part is the working area), (**e**) response(orange) and recovery(green) time, and (**f**) relative resistance in water.

Figure 5a,b shows the electrical signal response for small strains (1%, 2%, 3%, and 5%) and large strains (10%, 20%, 30%, and 50%). When the sensor was subjected to small strains, the GF decreased as the strain increased, and when the tensile strain was 2%, the GF was 42. When the sensor was subjected to a large strain, the GF was stable at 32 for tensile strains of 10% and 20%. The GF decreased to 28 for tensile strains of 30%, but overall the relationship was still linear, which corresponds to Figure 5c,d. The main reason for the sensor’s high sensitivity in the strain range is that there were many broken CNCs on the cracks of the sensing layer on the crack surface and the crack wall, which provided many contact points and significantly changed the relative resistance. However, the length of the broken CNCs was limited, and as the stretch increased, the contact points gradually decreased, thus, the sensitivity decreased.

Stepwise stretching was performed to test the resistance change under different stretching levels. Starting from the initial position, 3% stretching was applied each time and held for 3 s. This testing was repeated 10 times to observe the strain sensor’s resistance change. Figure 5c shows the results. It was observed that with the step change of strain, the sensor’s resistance also exhibited a step change, which can distinguish different strains in detail, reflecting the high sensitivity and stability of the sensor during the strain process. The overshoot and subsequent relaxation in the ΔR/R_0_ response were attributed to the viscoelastic nature of the polymer matrix and the interaction with the CNCs. During stepwise stretching, the polymer chains in the PDMS matrix experience delayed elastic recovery, leading to temporary resistance overshoot. This effect becomes more pronounced with increasing strain due to stress relaxation and polymer chain reconfiguration. Additionally, CNCs with high elasticity may shift within the polymer matrix under tensile stress and are unable to immediately return to their original position upon strain release, further contributing to the observed relaxation. Notably, PDMS is known to exhibit stress relaxation in a logarithmic fashion, irrespective of the presence of fillers [46]. Such hysteresis behavior is common in nanocomposites with nanoscale conductive networks, as reported in related studies [47,48,49].

Through Figure 5d and our calculations, it was found that the sensor operated in the interval of 0–40%, with a maximum gauge factor (GF) of 34 and a linear fit coefficient of R² = 0.98 in the 5–35% stretch interval. The CNCs have a certain degree of toughness and length, which plays a role in bridge grafting and the expansion of crack formation. However, when the stretching is too large, making the cracks too large, the CNCs disconnect at the cracks due to their limited length, gradually decreasing the relative resistance and finally converging to a constant value.

Figure 5e shows the sensor’s response and recovery time. The sensor was quickly stretched by 0.5% at a rate of 300 mm/min, held for 1 s, and then recovered at the same rate. From the testing, a sensor response time of 110 ms and a recovery time of 160 ms was obtained, ensuring the accuracy and real-time performance of the monitored results.

Figure 5f shows the change in relative resistance when the sensor was placed in water and removed from water. The relative resistance dropped slightly due to the water pressure on the sensing layer and recovered when removed from the water, reflecting the hydrophobicity of the sensor.

**Figure 6:**
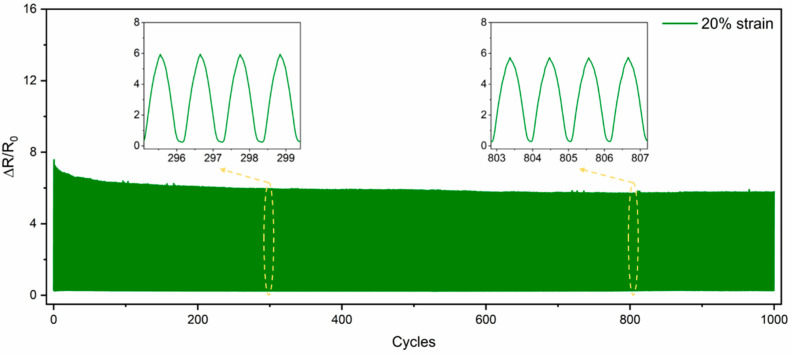
Cyclic test at 20% stretch for 1000 cycles.

Figure 6 shows the durability and stability of the CNCs/TPU sensors. The sensor was tested with 1000 cycles of 20% stretch at a rate of 90 mm/min for about 90 min to check the repeatability of its sensing behavior. Throughout 1000 cycles, no significant difference in the relative resistance was observed between stretching and recovery. Extracting the insertion sequence from the curves showed that the relative resistance always stayed around 6.0. The results show that the sensor is durable and stable during long-term use due to the strong adhesion between the conductive CNC and TPU nanofiber layers.

### 3.3. Hydrophobic Permeability

**Figure 7:**
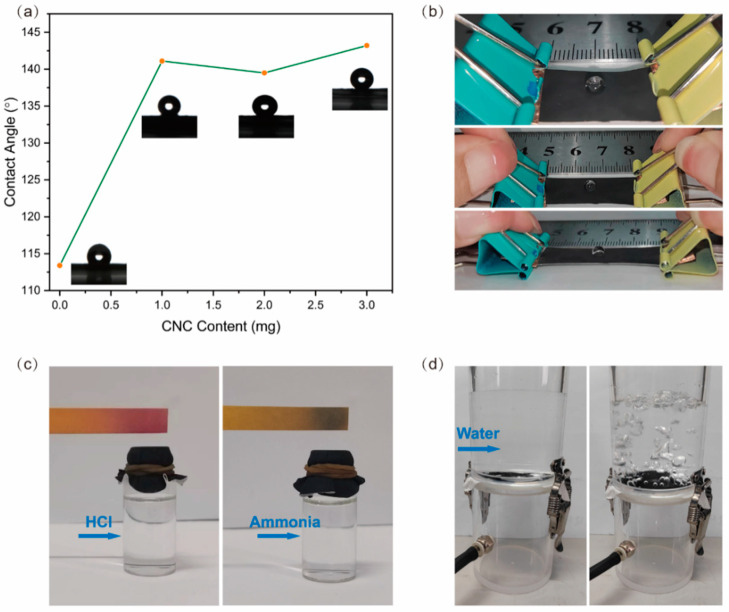
(**a**) Hydrophobic angle deposited by different contents of CNCs, (**b**) still good hydrophobicity after stretching, (**c**) pH paper changes color, (**d**) hydrophobic breathability experiments.

Considering the breathing problem of human skin when wearing flexible strain sensors, hydrophobic breathability experiments were conducted on the sensors. By depositing CNCs/PDMS on the surface of the TPU nanofiber film, the surface properties of the film were significantly altered. Figure 7a shows the CNCs/PDMS deposition variation versus the water contact angle (WCA). With the deposition of CNCs, the WCA of the film surface increased from 113.2° to 141.6°, and with the continued increase of CNCs, the WCA did not change much, with a maximum of 143.5°.

Figure 7b shows the hydrophobic performance of the sensor under different stretching. The sensor, with an original length of 2 cm, still had high hydrophobicity after stretching 50% and 100%. Figure 7c shows the permeability of the film. To test the gas permeability, the bottle was filled with a volatile acid (HCl) and alkaline (ammonia), secured with film and a leather strap, and the gas was tested using PH paper. A corresponding change in the pH paper was observed. Figure S5 and Video S1 show the rate of change of the pH test paper. Figure 7d shows the hydrophobicity and permeability of the film through a simple device. To test breathability, the film was sandwiched between two acrylic cylinders, with a certain amount of water placed on top and an airbag attached to the bottom for blowing air. The left image shows that the water was completely isolated on top of the film and was not dripping. After blowing air through the film, the right-side membrane exhibited an upward convex shape, accompanied by numerous bubbles floating from the film’s surface, reflecting the film’s hydrophobicity and breathability, as demonstrated in Video S2.

These experiments demonstrate that the sensor is hydrophobic and breathable, aligning with the biological characteristics of human skin. Therefore, it will not feel uncomfortable even if the sensor is attached to the skin for a long time.

Table 1 lists the performance, maximum strain, GF, and characteristics of CNCs/TPU strain sensors and compares them with other reported composites. Since most human joint movements are within the 50% strain range [50,51], we compared GF within the 50% strain range. Compared with other work using CNTs, rGO (graphene oxide), or CB (carbon black) as conductive materials, and PDMS or PLC (Polycaprolactone) as substrates, our strain sensors prepared using CNCs exhibit certain limitations in terms of strain range, yet demonstrate high sensitivity within that range. CNCs/TPU sensors are also sufficient for the detection of human motion. In addition, the sensor is also hydrophobic and breathable, further enhancing its suitability for use by people and its application in complex environments.

### 3.4. Application of Sensors in Human Motion Monitoring

The flexible strain sensor has excellent flexibility, high sensitivity, long-term stability, and good sensing performance. In addition, preparation of the sensor is straightforward and efficient, and its size can be customized according to specific requirements. The sensors-based wearable device has great potential for application in human motion monitoring.

**Figure 8:**
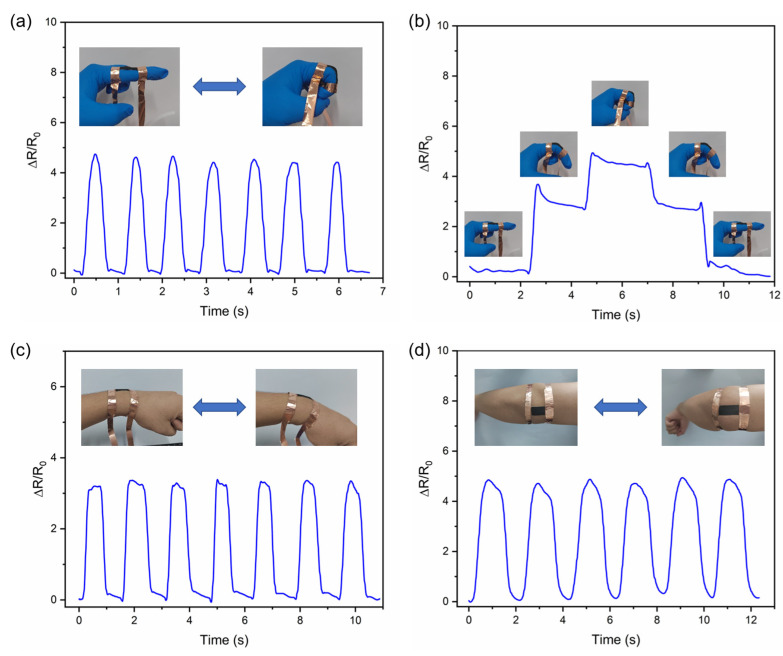
Sensor for human detection. (**a**) Finger flexion, (**b**) finger flexion at 45°, finger flexion at 90°, (**c**) wrist flexion, (**d**) elbow flexion.

In Figure 8a,b, the sensor was mounted on a finger joint, and the relative resistance of the sensor gradually increased as the finger bent, returning to the initial position when the finger straightened. When the finger was bent at 45° and 90°, the sensor’s relative resistance curve also increased stepwise with the angle change, demonstrating the sensor’s high sensitivity and ability to detect minor differences. When the sensor was mounted on the wrist, as shown in Figure 8c, the sensor provided a regular and accurate electrical signal response to wrist flexion. Mounting the sensor on the elbow joint resulted in a more significant relative resistance change and cycle time due to greater deformation of the elbow joint compared to the wrist joint. This is also consistent with the data curves in Figure 8d, making it easier to distinguish between wrist and elbow movements. The finger-bending experiment was also performed in water, and the results are presented in Figure S6.

**Figure 9:**
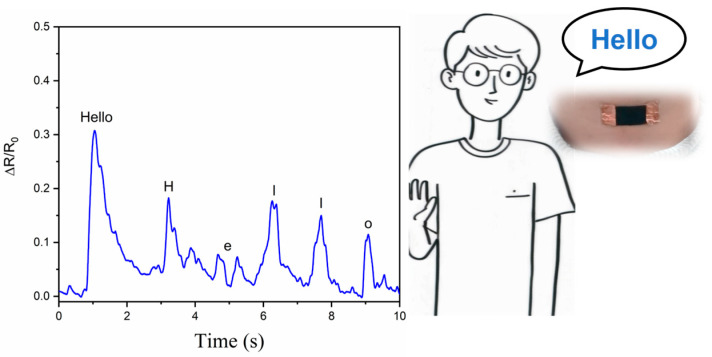
Detection of skin stretching and vibration during speech.

The sensor was attached to the neck at the laryngeal node position to detect stretching and vibration of the skin during speech. As shown in Figure 9, when we said “Hello” and each letter, the sensor detected a corresponding change in relative resistance. One of the more unusual instances was saying the letter “e”. When we say “e”, we usually only stretch the lips wider and rarely stretch the skin at the neck, resulting in a low change in relative resistance.

These results show that the sensor has high sensitivity and stability, which can meet different sensing scenarios and demands, with broad application prospects in flexible wearable devices.

## 4. Conclusions

In summary, we used electrostatic spinning and vacuum filtration to prepare a flexible strain sensor easily and efficiently. The sensor comprises a conductive CNCs/PDMS layer and a flexible TPU substrate. The PDMS layer significantly improves interfacial bonding between the CNCs and the TPU film, ensuring the sensor’s stability and durability in tensile, torsion, and fatigue tests. The three-dimensional helical structure of the CNCs and the porous structure of the TPU film provide the sensor with hydrophobicity and breathability, which increases comfort while wearing. Finally, performance tests and signal detection on different human body parts verified the sensors’ excellent performance and practical value. The flexible strain sensor is expected to achieve broad application prospects in fields such as smart wearable devices, intelligent robots, and the medical field.

## Figures and Tables

**Table 1:** Comparison of the sensing performance and functionality of flexible strain sensors.

Sensor Materials	Maximum Strain Range	GF Within 50% Strain	Characteristics	Reference
CNTs-CNCs/PDMS	9–260%	4.5–70		[40]
CB/MWCNTs/ TPU	0–50%	9–22		[52]
MWCNTs/TPU	300%	5.39		[53]
PCL/Graphene	200%	6.07		[54]
PDMS/MWCNTs	200%	30		[55]
MXene/CNCs	77%	58		[56]
CNT/F-TPU	550%	1.6	Superhydrophobic	[57]
CNTs/rGO TPU	100%	21	Breathable	[58]
CNCs/TPU	40%	34	Hydrophobic and breathable	This work

## Data Availability

Data that support the findings of this study are available from the corresponding author upon reasonable request.

## Supplementary Materials

The following supporting information can be downloaded at: https://www.mdpi.com/article/10.3390/nano15060457/s1, Figure S1: (a) Mass synthesis of CNCs and (b) TEM of CNCs; Figure S2: (a) Cross-section of the sensor and (b) enlargement. (c) Cross-section of the sensor in case of PDMS overdose; Figure S3: CNCs/PDMS XPS analysis; Figure S4: CNCs Connection Cracks; Figure S5: Rapid color change of PH test paper in 2s; Figure S6: Comparison of finger bending experiments in air and water.
