# Supplementary material for: Preparation and Application of Hydrophobic and Breathable Carbon Nanocoils/Thermoplastic Polyurethane Flexible Strain Sensors
Source: Nanomaterials (Basel). 2025 Mar 17;15(6):457. doi: 10.3390/nano15060457 (PMC11944968; doi:10.3390/nano15060457)
Supplement: Supplementary file 1 [file nanomaterials-15-00457-s001.zip › Supporting Information.pdf]

Supporting Information

Preparation and Application of Hydrophobic and Breathable Carbon Nanocoils/Thermoplastic Polyurethane Flexible Strain Sensors

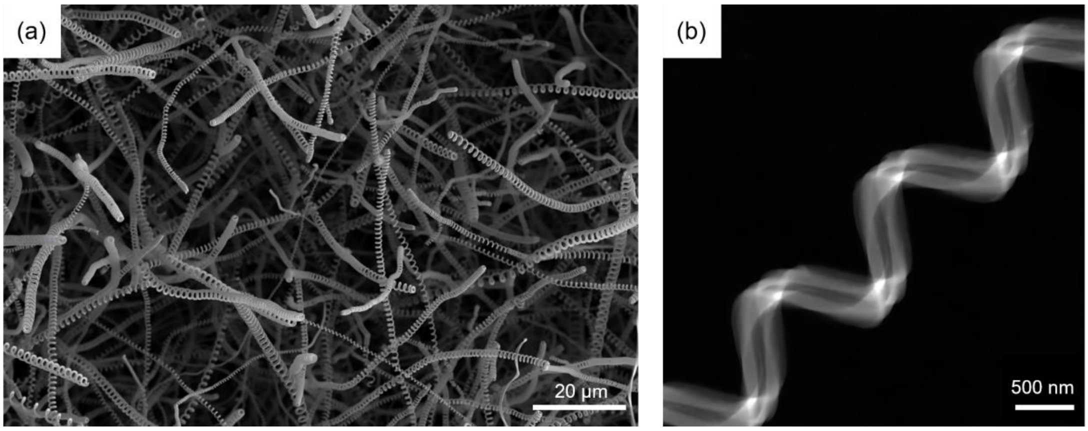

Figure S1. (**a**) Mass synthesis of CNCs and（**b**）TEM of CNCs.

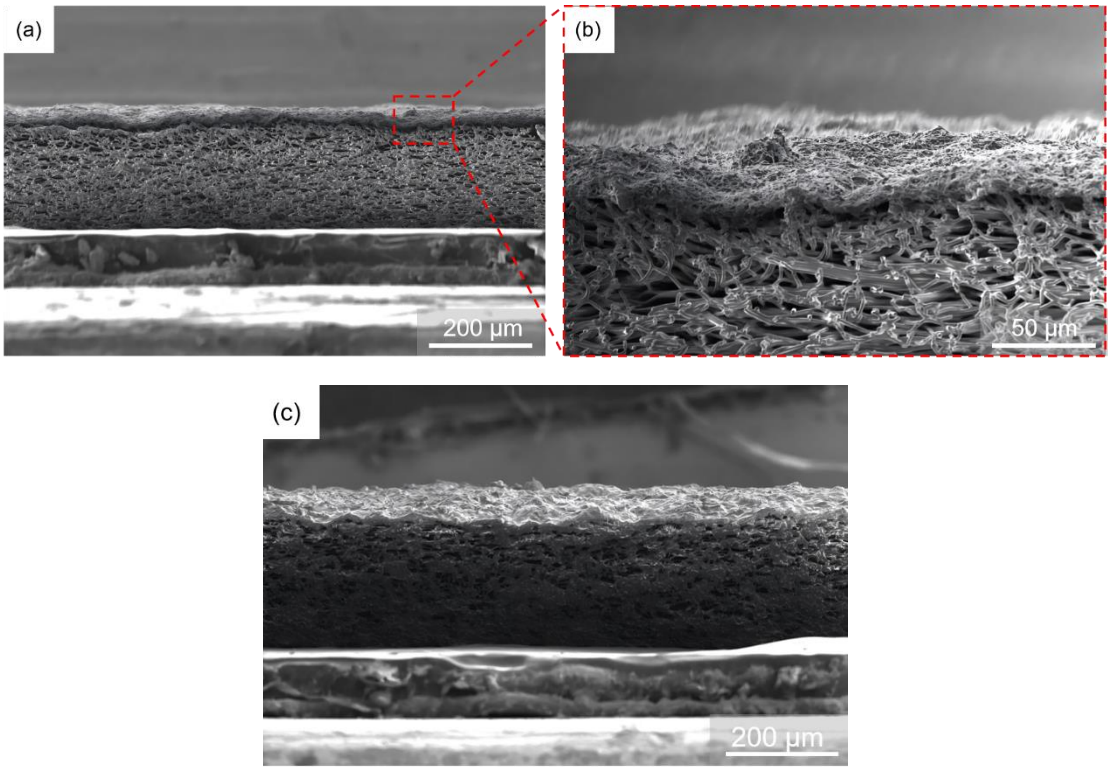

Figure S2. (**a**) Cross-section of the sensor and (**b**) enlargement. (**c**) Cross-section of the sensor in case of PDMS overdose.

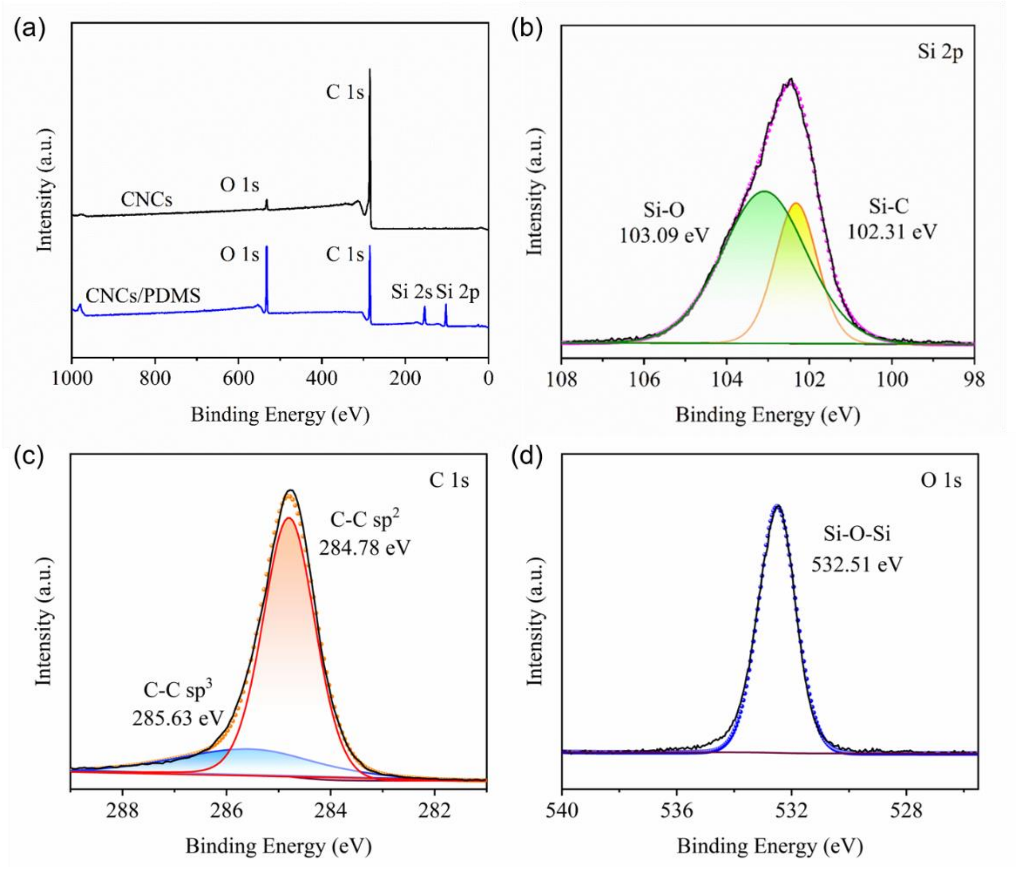

Figure S3. CNCs/PDMS XPS analysis.

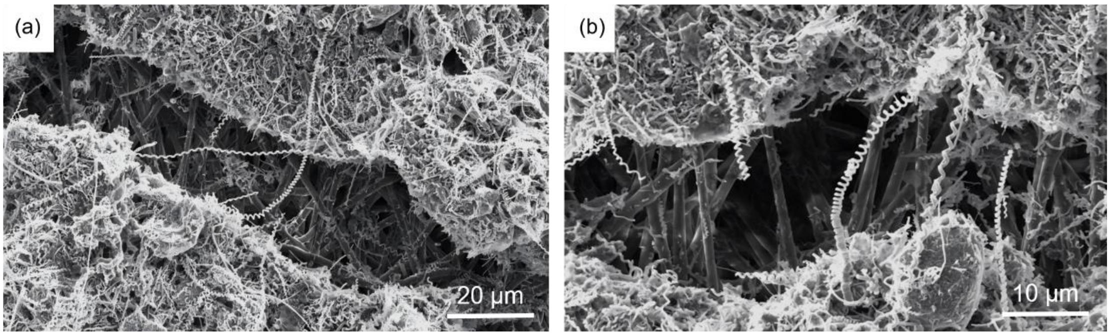

Figure S4. CNCs Connection Cracks.

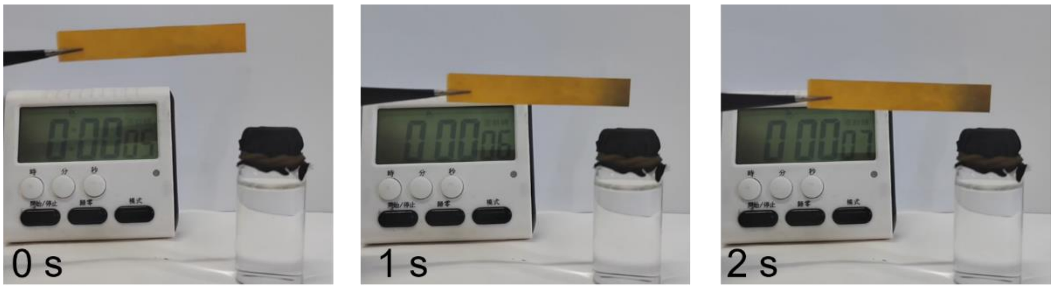

Figure S5. Rapid color change of PH test paper in 2s.

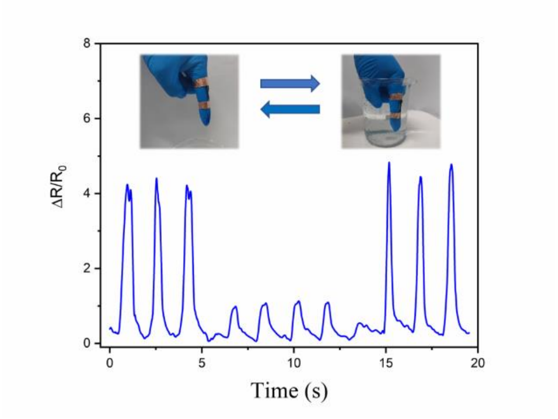

Figure S6. Comparison of finger bending experiments in air and water.
